# Individual performance in team-based online games

**DOI:** 10.1098/rsos.180329

**Published:** 2018-06-20

**Authors:** Anna Sapienza, Yilei Zeng, Alessandro Bessi, Kristina Lerman, Emilio Ferrara

**Affiliations:** 1 USC Information Sciences Institute, Marina del Rey, CA 90292, USA; 2 USC Department of Computer Science, Los Angeles, CA 90089, USA

**Keywords:** HCI, collaborative environments, online games, human performance

## Abstract

Complex real-world challenges are often solved through teamwork. Of special interest are ad hoc teams assembled to complete some task. Many popular multiplayer online battle arena (MOBA) video-games adopt this team formation strategy and thus provide a natural environment to study ad hoc teams. Our work examines data from a popular MOBA game, League of Legends, to understand the evolution of individual performance within ad hoc teams. Our analysis of player performance in successive matches of a gaming session demonstrates that a player’s success deteriorates over the course of the session, but this effect is mitigated by the player’s experience. We also find no significant long-term improvement in the individual performance of most players. Modelling the short-term performance dynamics allows us to accurately predict when players choose to continue to play or end the session. Our findings suggest possible directions for individualized incentives aimed at steering the player’s behaviour and improving team performance.

## 1. Introduction

Solving today’s complex challenges increasingly calls for collaborating with others. People are often brought together in temporary ad hoc teams to achieve a common goal before moving on to the next problem, likely with a different team. An example of such ad hoc teams can be found in multiplayer online battle arena (MOBA) games. In this popular genre of games, two teams are assembled and face each other, with individuals collaborating with strangers to complete a series of complex, fast-paced tasks (e.g. kill enemies, destroy towers and conquer the enemy base) to win the game.

Previous studies [1] showed that strangers collaborate in online games through communication and coordination, often trying to exert influence over their teammates. Players understand that the way they interact with teammates affects collaboration, and thus they must discipline themselves to facilitate successful social interaction with their team. Players must reach mutual understanding of the changing situations, work closely, continuously make new strategies together, build and maintain team cohesiveness, and deal with deviant players. In addition, game designers dynamically assemble players to match the skill levels of opposing teams. There are several factors that affect the ad hoc team performance, such as communication [2], social ties [3], composition [4,5], etc.

However, the performance of individuals within teams, and of the teams themselves, may evolve over time, as individuals improve and perfect their skills or learn how to work with others on a given shared task. Understanding how individual and team performance change over time can then provide suitable insights on how to assemble successful teams. To this aim, we study the performance of players in League of Legends (LoL), a popular MOBA game.

Data from MOBA games like LoL enable us to explore the following four research questions:

RQ1 Do players improve over time, as they acquire skills and experience through teamwork?
RQ2 Are there notable changes in individual performance during the course of a single team-playing session?
RQ3 If performance does change over a session, does experience mitigate its variation?
RQ4 What factors predict a player’s choice to continue playing or end a given session?

The data we study contain records of nearly 242 000 solo-queue matches played by 16 665 of the most active LoL players. After segmenting matches by sessions—periods of game play activity without an extended break—we track the player’s performance over the course of the session. We measure performance at two levels: the overall team’s performance and the individual player’s performance. The former is defined as the fraction of matches during a session won by the player’s team. The latter is defined on the basis of three main players’ actions during the game: the number of kills (K), the number of assists (A) and the number of deaths (D). We compute the kill–death–assist (KDA) ratio of the player, which is a value commonly used by players to compare their performance. Interestingly, both measures show that performance generally declines over the course of a single game playing session. This is surprising for two reasons: first, players in solo-queue matches do not choose their teammates in the game (we indeed consider this type of match to avoid the possible influence of playing with friends); second, the game is designed to match opposing teams’ skills and yield an equal probability of winning to each team. However, we systematically observe that the team to which a player is assigned wins on average fewer matches if that player had already played other matches without taking a break. While similar short-term performance deterioration was observed in the context of different online activities, such as commenting on Reddit [6] or Twitter [7], this is the first time that depletion effect was observed in the context of teamwork and in particular in online games. Moreover, we find that deterioration is more pronounced for novices, rather than veteran players, potentially reflecting the benefits of experience and learning within the game. To identify features predictive of the player’s behaviour, we train a classifier to predict whether the player will end the gaming session after the current match. We consider different sets of features related to various aspects of the game: match information, actions carried out by the player in the game and features related to their performance. We find that the most predictive features correspond to how many matches the player played in the current session and the win rate of the player both in the last match and throughout the session.

## 2. Data and methods

### 2.1. League of Legends and data collection

League of Legends is a multiplayer online game that combines elements of role-playing, real-time strategy and tower defence game genres. A single match consists of a strategic, fast-paced battle between two teams composed of five people, who are usually strangers. A team wins by destroying the opposing team’s nexus, a large structure fortified by defensive towers. While the destruction of the enemy nexus is the main goal, teams also aim to fulfill subgoals, which may be necessary for or conducive to victory; individual players also strive to achieve personal goals, such as a high kill/death ratio.

We collected data about LoL by using the LoL’s Riot Games API.^1^ With the aim of studying individual performance, we collected information of solo-queue matches, in which players cannot select their teammates. These specific matches allow us to avoid any influence that playing with friends might have on the final performance of players. We additionally require that each player in the dataset has at least 10 matches for two main reasons. First, we want to avoid biases related to players that try the game a few times and never play again. Second, we will focus our analysis on performance evolution in gaming sessions (as described in the following). Thus, we need each player to play at least few sessions in their history. The final dataset [8] consists of about 242 000 solo-queue matches played by a sample of 16 665 players between May 2014 and January 2016. The data contain information about matches, including match time and duration, and the number of deaths, kills, earned gold and gold spent for each player in each match. We reported some additional information about the dataset, such as the number of matches and sessions per player, average match durations, etc., in table 1.

**Table 1.** Dataset statistics summary. (#Match=242 352, #player= 16 665) The match duration, total play time/player and session play time/player in the table are displayed in minutes.

	# sessions per player	# matches per player	match duration	total play time per player	session play time per player
Min	1.0	1.0	5.2	61.2	5.2
Avg	132.0	239.7	33.8	965.7	52.0
Max	1312.0	1835.0	84.0	63 662.7	753.1

### 2.2. Gaming sessions

To address **RQ2**–**RQ3**, we will need to identify sessions of continuous player activity. Time series of a player’s matches can be decomposed into gaming sessions, i.e. periods of activity without an extended break. The sessions can be identified by examining time intervals between consecutive matches. Cases where this interval exceeds some predefined threshold are used to separate matches into different sessions [9,6]. Here, we define a gaming session of length *n* as the temporally ordered sequence of *n* matches, with no more than a 15-minute break between matches. The break length, corresponding to the median of the distribution of break times between matches, is computed over the most active players of our dataset (i.e. players having at least 10 matches in their history).

**Figure 1.**
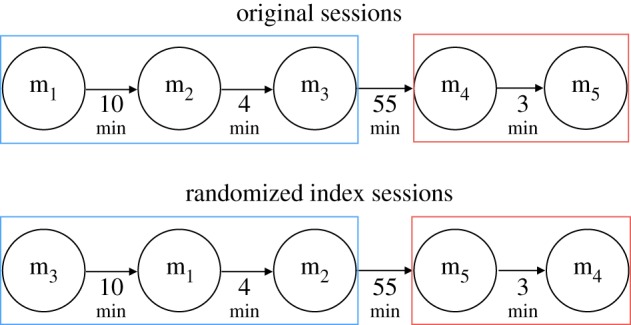
Original sessions and randomized index sessions.

To check the robustness of our findings regarding individual performance and verify that they are not due to chance, we also carry out an analysis of randomized session data, i.e. sessions where the order of matches for individual players was randomly shuffled according to the strategy depicted by figure 1. The results of this test will be presented later (see §3.2).

### 2.3. Prediction methods

To address **RQ4**, in our analysis we will present a prediction task that will leverage the three methods described as follows.

*Random forest* is an ensemble-based learning method for classification and prediction that operates by constructing a multitude of decision trees at training time and outputs the class that is the mode of the classes or mean prediction of the individual trees [10]. Random forests increase generalization accuracy of decision tree-based classifiers without compromising accuracy on training data [11]. In particular, random forests correct for the problem of decisions trees over-fitting to the training data [12].

*Gradient boosting* is a machine learning technique which produces a prediction model in the form of an ensemble of weak prediction models, typically decisions trees. Gradient boosting produces competitive, highly robust, interpretable procedures for both regression and classification [13].

*Adaptive boosting* is a machine learning meta-algorithm which produces a prediction model combining weak learners (typically decision trees) into a weighted sum that represents the final output of the boosted classifier [14,15]. The term *adaptive* means that subsequent weak learners are adjusted in favour of those instances misclassified by previous classifiers. Even if such an approach is sensitive to noisy data and outliers, as long as the performance of each weak classifier is slightly better than random guessing, the final predictive model can be proved to converge to a strong learner [12].

Moreover, for each classification method, we learn three models, in which we incrementally add different sets of features: (i) match metadata, such as *player id*, *match position in a session* and *match duration*; (ii) player’s actions, such as *kills*, *deaths* and *assists*; and finally, (iii) player’s performance measures, such as the *KDA* and the binary information about whether the *player wins* in the match or not, etc.

## 3. Results

In this paper, we study the performance of a set of LoL players who played at least 10 solo-queue matches. We require at least 10 matches to consider players who engaged in the game long enough to play a few sessions in their history, and avoid the bias that might occur when considering players that try the game a few times and quit. Importantly, we only select solo-queue matches, in which players cannot decide their team, or part of their team, thus avoiding possible influences of friends in the game.

Our dataset is then composed of about 242 000 matches played by 16 665 different players. In the following, we will address the research questions previously defined, and we will provide some insights of the possible mechanisms underlying our observations.

### 3.1. RQ1: long-term performance

**Figure 2.**
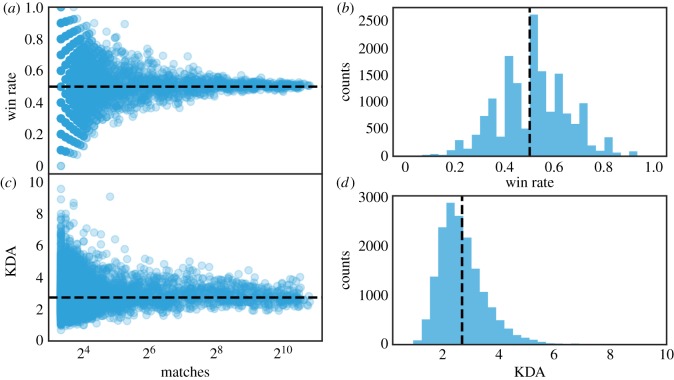
Relationship between experience and player performance.

First, we examine how performance changes with experience (**RQ1**), thus we compute long-term performance of players by taking into account their entire history in the dataset, i.e. the total number of matches of each player. Here, we consider two measures of performance. First, we define a team performance measure, which is computed as the fraction of wins. Second, we define an individual performance measure, namely the kill–death–assist ratio KDA, defined as (k+a)/max(1,d), where *k* is the number of kills, *a* is the number of assists and *d* is the number of deaths of a player in a given match. Figure 2 reports how performance changes, measured by the overall fraction of wins (*a*,*b*) and KDA (*c*,*d*) for each player as they play more matches. As we can observe, there is no long-term team’s performance improvement with experience (*ρ*=0.02). The longer the users play, the more the performance related to their teams reverts to the mean—which is approximately 0.5 (figure 2*a*,*b*). A possible explanation might be related to the design of the game. In fact, players are given Elo-like ratings—a method used to calculate the relative skill of players in competitor-versus-competitor games such as chess—and these ratings are used to assemble teams of players with comparable skills. In other words, if a player’s skill improves he/she will be paired up against players with similar skill level, and analogously if the skill level decreases. Thus, the likelihood to win each match is not significantly better than 50%. We noted the same effect when studying the KDA ratio, whose values revert to the mean score of 2.7 (figure 2*c*,*d*).

### 3.2. RQ2: short-term performance

**Figure 3.**
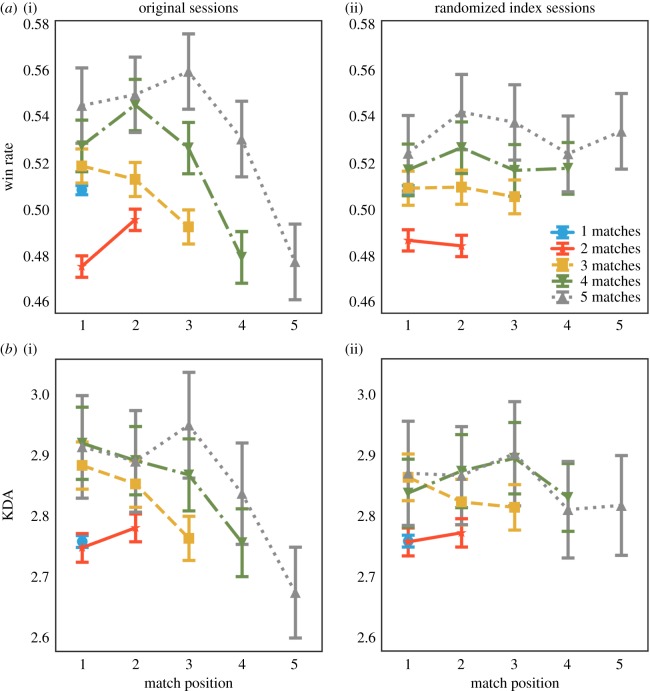
Performance deterioration over the course of a gaming session. Each line reports average (*a*) win rate or (*b*) KDA ratio for each successive match of a gaming session of a given length. Matches played later in the session have lower performance (left plots), but not when play data have been randomized (right plots). Error bars represent standard deviations (standard errors would be almost invisible due to large sample sizes). (*a*) Win rate and (*b*) KDA ratio.

Our second question (**RQ2**) explores short-term performance over the course of one session. In contrast to long-term performance, player’s performance, measured by both the fraction of matches the player’s team won and the player’s KDA of each match, degrades measurably over the course of a single session. Figure 3*a*(i),*b*(ii) provide a comparison between the performance achieved by players in sessions of different length (number of matches going from 1 to 5). We can observe that both types of performance at the end of a session are lower than at the beginning of that session. Moreover, the longer the session, the larger the performance decline: for sessions with three or more matches, the win rate and the KDA value, respectively, deteriorate by more than 10% and 8% between the first and the last matches in the session. Such short-term performance deterioration is not present in the randomized data (figure 3*a*(ii),*b*(ii)), suggesting the presence of a real effect and not simply a byproduct of data heterogeneity.

Performance declines over the course of a session according to both measures (win rate and KDA). The only difference is the initial improvement during longer game playing sessions: this pattern might reflect a ‘warm-up’ period. This pattern is stronger for the team’s performance measure (win rate) than for the player’s performance measure (KDA). The decline in team’s performance suggests that the teams a player is assigned to later in the session do not perform as well as the teams the player is assigned to earlier in the session. On the other hand, deterioration is also observed in individual performance. This phenomenon might be associated with some cognitive effect, such as mental fatigue, boredom or attention decline (we report relevant research in this area in §4).

### 3.3. RQ3: effect of experience on performance deterioration

**Figure 4.**
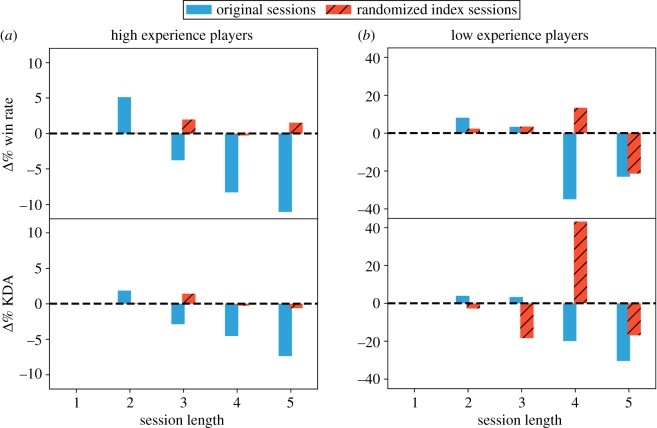
Comparison of performance deterioration in high versus low experience players. (*a*) high experienced players’ (top 5 percentile) win rate and KDA performance comparison and (*b*) win rate and KDA performance comparison for low experienced players (bottom 5 percentile).

Does experience mitigate performance declines? To answer our third research question (**RQ3**), we studied how deterioration is linked to players’ experience. To this end, we ranked players by the number of matches they played and compared highly experienced players (those in the 95th percentile or above) with the less experienced players (those below the 5th percentile by number of matches played). Figure 4 shows the magnitude of performance deterioration over the course of sessions played by the highly experienced players (*a*) and the less experienced ones (*b*). Performance of the latter group of players declines far more than that of the experienced players. Comparison to randomized data suggests that these trends are not due to chance.

**Figure 5.**
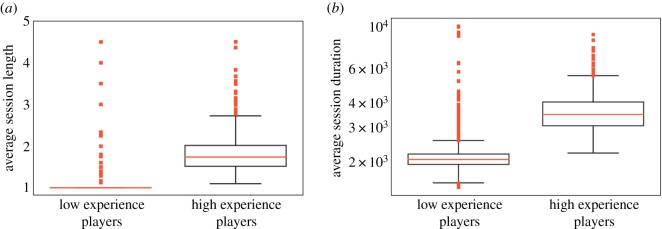
Comparison of highly experienced versus inexperienced players. (*a*) Average session length and (*b*) session duration (in seconds) for these players.

This suggests that player experience mitigates the mechanisms that lead to short-term deterioration of performance. For example, experienced players may use their available cognitive resources more efficiently and stretch them over more games. Analysis provides some support for the hypothesis that highly experienced players tend to engage in longer gaming sessions compared to the less experienced players. Boxplots in figure 5*a* show that the average length of sessions played by these two groups of players is significantly different (Wilcoxon test, *p*<0.0005). The difference is still statistically significant even when only the player’s first 20 sessions are taken into account (Wilcoxon test, *p*<0.0005), indicating that highly experienced players are different from other players already at the beginning of their tenure. These players not only play more games during a session, they also play for longer. Boxplots in figure 5*b* show that the duration of sessions (in seconds) of the highly versus less experienced players are also significantly different (Wilcoxon test, *p*<0.0005). Although the reason why the more experienced players are able to play longer is still unknown, its net effect is to partially shield these players from the effects of performance deterioration.

### 3.4. RQ4: short-term engagement prediction

To address our last question (**RQ4**), we focus on player engagement. In particular, we examine what characteristics predict if some players engage with short gaming sessions while others go on to have longer sessions. We formulate this problem as a prediction task. Specifically, given a player’s history, described by a set of match-related features, our goal is to predict whether a given match will be the player’s last in the session. We chose three different sets of features to characterize players: features describing *matches*, *game actions* and *performance*. Match features (henceforth, MATCH) include:

— match: current match’s position in the current session;
— match duration: duration (in seconds) of the current match;
— cumulated match duration: duration (in seconds) of the current session.
— mean match duration: average match duration in the current session;
— sessions: total number of sessions played until now;
— player id: the unique identification of each player;
— experience: total number of matches played until current match.

Players’ actions (henceforth, ACTIONS) in the game include:

— kills: number of kills a player performed in the current match;
— deaths: number of deaths a player suffered in the current match;
— assists: number of assists a player carried out in the current match;
— cumulated kills: total number of kills a player performed in the current session;
— cumulated deaths: total number of deaths a player suffered in the current session;
— cumulated assists: total number of assists a player helped in the current session;
— mean kills: average kills a player performed per match in the current session;
— mean deaths: average deaths a player suffered per match in the current session;
— mean assists: average assists a player carried out per match in the current session.

Finally, we characterize players’ performance (henceforth, PERFORMANCE) through the following features:

— KDA: kill–death–assist (KDA) ratio of a player in the current match;
— cumulated KDA: KDA ratio of a player in the current session;
— mean KDA: average KDA a player achieved per match in the current session;
— win: binary variable indicating whether the player won or lost the current match;
— session win rate: fraction of wins in the current session;
— current win rate: fraction of wins until the current match in the current session.

We label each match in the dataset as a *positive* outcome if that match is the last match of the player’s session, and a *negative* outcome if the player keeps playing after that match. Our dataset is mildly unbalanced, containing 145 169 positive labels and 261 037 negative ones. This is consistent with the presence of several sessions of length greater than 1 (i.e. with at least two matches). In machine learning, standard evaluation metrics that do not account for uneven class distribution can be misleading. To address this challenge, we perform two different predictive tasks: (i) we use the full (unbalanced) dataset to evaluate the performance of three prediction models by means of the area under the receiving operator characteristic curve (AUC), providing an evaluation for the true and false positive rates of the model predictions (where *AUC*=1 represents a perfect test); (ii) we under-sample the original data to obtain a balanced dataset and evaluate the performance of our prediction models through standard metrics such as precision (i.e. the fraction of true predicted positive outcomes over all positive predictions), recall (i.e. the fraction of true predicted positive outcomes over all positive outcomes), accuracy (i.e. the fraction of correctly predicted outcomes over all outcomes) and F1 (which combines precision and recall measures).

In both prediction tasks, we compare the performance of three ensemble-based prediction models: random forest (RF), gradient boosting (GB) and adaptive boosting (AB). To find the best combination of hyper-parameters, for each classifier we perform a 10-fold cross-validated grid search over the hyper-parameters’ space. To prove robustness of results, we report mean scores and standard deviations obtained via Monte Carlo cross validation. Here, we use 90% of the data samples to train and the remaining 10% to test our models.

For each classification algorithm (RF, GB and AB), we learn three distinct predictive models in which we cumulatively add the different sets of features: (1) we only consider match metadata (namely, MATCH); (2) we additionally take into account the action features (namely, MATCH + ACTIONS); and finally (3) we add the features related to performance (namely, MATCH + ACTIONS + PERFORMANCE). This procedure is commonly called *model nesting*.

In the first prediction task (unbalanced data), the best performance is obtained by model 3 where all the 22 features are used (i.e. MATCH + ACTIONS + PERFORMANCE). As shown in table 2, the best result is obtained by GB (*AUC*=0.976±0.001), followed by RF (*AUC*=0.968±0.001 over 512 different decision trees), and AB (*AUC*=0.914±0.002). The most significant features, whose Gini index (i.e. a score indicating the relevance of each specific feature in the prediction task) is reported in table 3, used by the GB classifier are session win rate (feature *importance*=0.163), current win rate (feature *importance*=0.286) and match (feature *importance*=0.087). The importance of the match index in the session, which is an indicator of how much time players have already spent in the game, in predicting behaviour suggests that people have a finite budget—whether of time or cognitive resources—for game play. At the same time, the overall team performance (current and session win rate) also decreases during the session. The perception of decreasing win rate, combined with exhaustion of a finite budget, may lead to the player’s decision to quit the game.

**Table 2.** Classification performance metrics scores. The best model performances are highlighted in italics.

		model 1 (MATCH)	
	RF	GB	AB
AUC	0.830±0.003	*0.837*±*0.002*	0.837±0.003
F1	0.803±0.002	*0.818*±*0.002*	0.818±0.003
precision	*0.709*±*0.004*	0.702±0.003	0.701±0.004
recall	0.926±0.002	0.981±0.001	*0.982*±*0.001*
accuracy	0.773±0.002	*0.783*±*0.002*	0.783±0.003

		model 2 (MATCH + ACTIONS)	
	RF	GB	AB
AUC	0.827±0.003	*0.839*±*0.001*	0.836±0.002
F1	0.813±0.002	*0.819*±*0.002*	0.818±0.002
precision	0.703±0.004	*0.704*±*0.003*	0.701±0.003
recall	0.965±0.002	0.979±0.001	*0.981*±*0.001*
accuracy	0.779±0.003	*0.783*±*0.002*	0.782±0.003

		model 3 (MATCH + ACTIONS + PERFORMANCE)	
	RF	GB	AB
AUC	0.968±0.001	*0.976*±*0.001*	0.914±0.002
F1	*0.962*±*0.001*	0.959±0.001	0.888±0.003
precision	*0.927*±*0.002*	0.922±0.002	0.824±0.004
recall	*0.999*±*0.000*	0.999±0.000	0.962±0.003
accuracy	*0.960*±*0.001*	0.957±0.001	0.878±0.003

**Table 3.** Feature importance table. Ranking based on the Gini splitting index.

random forest		gradient boosting		adaptive boosting	
feature name	score	feature name	score	feature name	score
model 1 (MATCH)					
match	0.368	cum. match duration	0.249	experience	0.371
match duration	0.131	experience	0.183	session	0.354
player id	0.113	session	0.148	match duration	0.080
mean match duration	0.105	match duration	0.117	player id	0.076
experience	0.103	player id	0.112	mean match duration	0.063
cum. match duration	0.101	mean match duration	0.096	cum. match duration	0.043
session	0.079	match	0.095	match	0.014
model 2 (MATCH + ACTIONS)					
match	0.364	cum. match duration	0.141	experience	0.342
match duration	0.069	experience	0.141	session	0.334
player id	0.061	session	0.139	player id	0.063
experience	0.060	player id	0.091	match duration	0.059
mean match duration	0.054	match	0.080	mean match duration	0.039
session	0.046	match duration	0.078	cum. kills	0.023
cum. match duration	0.046	cum. kills	0.046	cum. match duration	0.021
mean assists	0.038	cum. deaths	0.045	cum. assists	0.021
assists	0.037	mean match duration	0.044	mean assists	0.020
mean kills	0.035	cum. assists	0.043	mean kills	0.016
mean deaths	0.034	assists	0.033	mean deaths	0.016
kills	0.034	kills	0.032	kills	0.012
cum. assists	0.033	mean deaths	0.025	cum. deaths	0.12
cum. kills	0.030	mean kills	0.024	match	0.008
deaths	0.030	deaths	0.020	deaths	0.008
cum. deaths	0.028	mean assists	0.020	assists	0.008
match	0.364	current win rate	0.301	session win rate	0.367
current win rate	0.335	session win rate	0.194	current win rate	0.209
session win rate	0.111	match	0.087	experience	0.135
match duration	0.020	cum. match duration	0.072	session	0.129
player id	0.018	experience	0.058	match duration	0.035
experience	0.016	session	0.051	cum. match duration	0.020
mean match duration	0.014	match duration	0.036	player id	0.018
KDA	0.013	player id	0.029	match	0.016
cum. match duration	0.012	mean match duration	0.022	KDA	0.012
session	0.012	cum. assists	0.021	mean match duration	0.010
mean KDA	0.010	cum. kills	0.021	deaths	0.008
cum. KDA	0.010	cum. deaths	0.017	mean deaths	0.008
assists	0.009	mean assists	0.014	assists	0.006
random forest		gradient boosting		adaptive boosting	
feature name	score	feature name	score	feature name	score
kills	0.008	KDA	0.013	mean assists	0.006
mean assists	0.008	cum. KDA	0.011	cum. kills	0.004
mean kills	0.008	mean KDA	0.010	cum. deaths	0.004
cum. assists	0.007	deaths	0.010	cum. assists	0.004
cum. kills	0.007	mean kills	0.008	cum. KDA	0.004
deaths	0.007	kills	0.008	mean KDA	0.004
mean deaths	0.006	assists	0.008	mean kills	0.002
cum. deaths	0.006	mean deaths	0.007	win	0.002
win	0.000	win	0.003	kills	0.000

In the second prediction task (balanced data), the highest accuracy is again achieved by model 3 (MATCH + ACTIONS + PERFORMANCE). The best results, shown in table 2, are provided by RF (*accuracy*=0.960±0.001), followed by GB (*accuracy*=0.957±0.001) and AB (*accuracy*=0.878±0.003). Consistently with the results provided in the first prediction task, the features identified by the RF classifier as most predictive are: match (feature *importance*=0.364), current win rate (feature *importance*=0.335) and session win rate (feature *importance*=0.111).

## 4. Related work

### 4.1. Individual and team performance in games

Various recent studies explored human performance and activity in online games. Several authors investigated aspects of team performance [2,4,5,16], as well as individual performance [17–21] in multiplayer team-based games. In Mathieu *et al*. [22], an extensive review about team effectiveness is provided. Here, the authors analyse different aspects of teamwork, such as team outcomes (team performance, members’ affect and viability), mediator–team outcome relationships and team composition.

Other aspects of social and group phenomena in virtual environments were covered in the review by Sivunen & Hakonen [23]. In this work, the authors identified four major topics related to virtual environment studies: testing that laws of social behaviours in real-life also apply in virtual environments, finding social behaviour norms, focusing on micro-level social phenomena, and filling the gap in well-established theoretical discussions and paradigms within social science.

The ‘optimal’ composition of temporary teams also attracted a lot of research: Kim *et al.* [4,5] studied LoL to determine how team composition affects team performance. Using mixed-methods approaches, the authors studied in-game role proficiency, generality and congruency to determine the influence of these constructs on team performance. Proficiency in tacit cooperation and verbal communication highly correlate with team victories, and learning ability and speed of skill acquisition differentiate novice from elite players. The importance of communication and its effects on team performance has been extensively studied by Leavitt and collaborators [2] once again in LoL: the authors studied both explicit and implicit (non-verbal, i.e. pings) communication, highlighting differences based on player styles, and different extents of effectiveness in individual performance increase.

Finally, the topic of individual performance in online games has been studied in different platforms. Shen *et al.* [24] suggested in their paper that gender-based performance disparities do not exist in massive multiplayer online games (MMO). In their work, the authors operationalized game performance as a function of character advancement and voluntary play time, based on Steinkuehler & Duncan [25] and show how character levels correlate with other types of performance metrics.

Other works looking at individual performance analyse first-person shooter games: Microsoft researchers studied the performance trajectories of Halo players, as well as the effect that taking prolonged breaks from playing has on their skills [17]. Analysing individual game performance allowed them to categorize players in groups exhibiting different trajectories, and then study how other variables (demographics, in-game activity, etc.) relate to game performance. This analysis reveals the most common performance patterns associated with first-person online games, and it allows to model skill progression and learning mechanisms. Finally, Vicencio-Moreira *et al*. [18] studied individual performance as a tool to balance game design and game-play: the authors defined several statistical models of player performance and associated them to multiple dimensions of game proficiency, demonstrating a concept of an algorithm aimed at balancing individual skills by providing different levels of assistance (e.g. aim assistance, character-level assistance, etc.) to make the game-play experience more balanced and satisfactory by matching players of different skill levels.

To the best of our knowledge, ours is the first study to focus on individual performance within temporary teams, to analyse the effect of performance deterioration over the short term, and to determine its interplay with engagement.

### 4.2. Team-based online games and engagement

Video-games represent a natural setting to study human behaviour. Prior to this study, several works have been devoted to analysing the behaviour and activity of players in multiplayer games. In particular, behavioural dynamics of team-based online games have been extensively studied in role-playing games like World of Warcraft [26,27], in battle arena games like League of Legends [1,19,28] and in other games [21,29,30].

The earlier studies focused on massively multiplayer online games like World of Warcraft, which exhibit both a strong component of individual game-play (e.g. solo quests aimed at increasing one’s character level and skills) as well as collaborative instances (e.g. raid bosses). First Nardi & Harris [26], and Bardzell and collaborators shortly after [27], analysed the five-person raid-boss instance runs to determine the ingredients of successful cooperative game-play. By means of a mixture of survey-based and data-driven analysis, the authors illustrated how the social component (i.e. chatting with teammates, and guild-based activity) was the leading factor to satisfaction and engagement.

Later studies focused on MOBAs: Kuo *et al.* [1,28] investigated engagement mechanisms on LoL by means of semi-structured interviews with players, aimed to unveil the elements behind successful team composition in temporary teams. Communication (written and oral) and effective collaboration strategies were linked to satisfactory game experience. Similar results hold for other MOBAs [29,30]. Concluding, a recent study investigated the relation between brain activity and game-play experience in multiplayer games: playing with human teammates yields higher levels of satisfaction but lower overall performance and coordination than playing with computer-controlled teammates [31].

Despite the fact that our work does not focus on the analysis of engagement in team-based online games, the results we found could be leveraged to design incentives to increase players’ engagement over time and used to prevent players from quitting the game.

### 4.3. Performance deterioration

Performance deterioration following a period of sustained engagement has been demonstrated in a variety of contexts, such as student performance [32], driving [33], data entry [34], self-control [35] and, more recently, online activity [7,6]. In particular, in *vigilance tasks*—i.e. tasks which require monitoring visual displays or auditory systems for infrequent signals—performance was shown to decrease over time, with concomitant increases in perceived mental effort [36]. For example, after long periods in flight simulators, pilots are more easily distracted by non-critical signals and less able to detect critical signals [37].

Factors leading to a deteriorating performance are still debated [38–40]. However, deterioration has been shown to be associated with physiological brain changes [41–43], suggesting a cognitive origin, whether due to mental fatigue, boredom or strategic choices to limit attention. In particular, mental fatigue refers to the effects that people experience following and during the course of prolonged periods of demanding cognitive activity, requiring sustained mental efficiency [41]. Persistent mental fatigue has been shown to lead to burnout at work, lower motivation, increased distractibility and poor information processing [41,44–50].

Moreover, mental fatigue is detrimental to individuals’ judgements and decisions, including those of experts—e.g. judges are more likely to deny a prisoner’s request as they advance through the sequence of cases without breaks on a given day [51], and evidence for the same type of cognitive fatigue has been documented in consumers making choices among different alternatives [52] and physicians prescribing unnecessary antibiotics [53]. Recent studies indicate that cognitive fatigue destabilizes economic decision-making, resulting in inconsistent preferences and informational strategies that may significantly reduce decision quality [54].

Short-term deterioration of individual performance was previously observed in other online platforms. It has been shown that the quality of comments posted by users on Reddit social platform [6], the answers provided on StackExchange question-answering forums [55], and the messages written on Twitter [7] decline over the course of an activity session. In all previously studied platforms, users worked individually to produce content or achieve some results, while in the present work, we considered both measures for individual performance (i.e. KDA) and the performance achieved by the team (i.e. win rate). We can interpret the KDA ratio of a player as the quality of his/her playing style during a match, and this can be compared to the results previously achieved in other types of platforms.

## 5. Conclusion

In this paper, we addressed four research questions concerned with modelling individual performance within temporary teams. To this aim, we studied players of a team-based online game, League of Legends, and measured performance at the level of the team, as the fraction of matches the player’s team won, and at the individual level, by computing the KDA ratio of the player at the end of each match. In the long term, we observed that there is no evident performance (both team and individual) improvement with experience and that both measures of performance are around their mean value. This observation might be linked to the game design: the team composition balancing strategy limits individual performance variance and thus reduces individual contributions to their team performance.

In the short term, i.e. over the course of a single game-playing session, our performance measures showed a strong deterioration pattern: the longer a player’s session is the more performance decreases, with metrics decreasing on average by 8–10% between the beginning and end of a session. Our findings are consistent with observations made on different online platforms and social networks, where performance deterioration was observed over the course of sessions. We found, however, that experience modulates short-term performance changes, potentially reducing the effects of performance depletion. Player experience (i.e. the overall number of matches played by each individual) appeared indeed to mitigate some of the effects of performance deterioration: the more experienced players showed less performance decline over the course of a game session than the less experienced ones. Other factors that were not investigated in the present work can influence performance in team-based games: the presence of friends in the team could trigger higher collaborative behaviour, players’ performance in the MOBA game can be also affected by the role the players are impersonating, and the composition of the team can have an effect on players decisions during the game.

We have shown, through the analysis of performance in the short term, that players tend to quit the game session after a certain number of matches in which their performance declines. We also investigated the factors that are predictive of a player quitting a game session. To this aim, we designed a prediction task in which we defined three sets of features. Each of these sets describes a specific aspect of the game. We took into account features related to matches, players’ actions and performance. We found that the features that best predict whether the player will quit the session are those associated with the match histories (session length, match duration, etc.). These findings are consistent with the hypothesis that players have a finite ‘cognitive budget’ for playing, which they deplete with game-play. While our work does not address the origins of depletion—whether through growing boredom or cognitive fatigue—we have shown that this phenomenon has different effects on experienced and inexperienced players.

By leveraging our findings, *individualized incentive strategies* could be designed to identify different classes of performers, and reward them dynamically and differently based on personalized, relative assessments of performance. This would allow to overcome the issues related to long-term performance and game design, by guaranteeing a satisfactory game experience for both experienced and inexperienced players. Moreover, incentives that enhance players’ engagement in the game could be used in combination with our predictions to prevent a player’s choice to quit the session, or frustration that may drive them to quit the game. Our future efforts will thus be devoted to further the research in the science of individualized incentives.

## Footnotes

1 Riot Games API: https://developer.riotgames.com/.

## Data accessibility

The Code used in this study is openly available at: http://www.emilio.ferrara.name/datasets The Dataset used in this study has been deposited in the Harvard Dataverse repository: http://dx.doi.org/10.7910/DVN/B0GRWX.
